# Virulence factors and molecular characteristics of *Shigella flexneri* isolated from calves with diarrhea

**DOI:** 10.1186/s12866-021-02277-0

**Published:** 2021-07-16

**Authors:** Zhen Zhu, Weiwei Wang, Mingze Cao, Qiqi Zhu, Tenghe Ma, Yongying Zhang, Guanhui Liu, Xuzheng Zhou, Bing Li, Yuxiang Shi, Jiyu Zhang

**Affiliations:** 1 Key Laboratory of New Animal Drug Project of Gansu Province, Key Laboratory of Veterinary Pharmaceutical Development of the Ministry of Agriculture, Lanzhou Institute of Husbandry and Pharmaceutical Sciences of CAAS, Jiangouyan, Qilihe District, 730050 Lanzhou, China; 2 College of Life Science and Food Engineering, Hebei University of Engineering, Hanshan District, 056038 Handan, China

**Keywords:** *S. flexneri*, Virulence factors, MLST, MLVA, PFGE

## Abstract

**Background:**

The natural hosts of *Shigella* are typically humans and other primates, but it has been shown that the host range of *Shigella* has expanded to many animals. Although *Shigella* is becoming a major threat to animals, there is limited information on the genetic background of local strains. The purpose of this study was to assess the presence of virulence factors and the molecular characteristics of *S. flexneri* isolated from calves with diarrhea.

**Results:**

Fifty-four *S. flexneri* isolates from Gansun, Shanxi, Qinghai, Xinjiang and Tibet obtained during 2014 to 2016 possessed four typical biochemical characteristics of *Shigella*. The prevalences of *ipaH*, *virA*, *ipaBCD*, *ial*, *sen*, *set1A*, *set1B* and *stx* were 100 %, 100 %, 77.78 %, 79.63 %, 48.15 %, 48.15 and 0 %, respectively. Multilocus variable number tandem repeat analysis (MLVA) based on 8 variable number of tandem repeat (VNTR) loci discriminated the isolates into 39 different MLVA types (MTs), pulsed field gel electrophoresis (PFGE) based on *Not*I digestion divided the 54 isolates into 31 PFGE types (PTs), and multilocus sequence typing (MLST) based on 15 housekeeping genes differentiated the isolates into 7 MLST sequence types (STs).

**Conclusions:**

The findings from this study enrich our knowledge of the molecular characteristics of *S. flexneri* collected from calves with diarrhea, which will be important for addressing clinical and epidemiological issues regarding shigellosis.

**Supplementary Information:**

The online version contains supplementary material available at 10.1186/s12866-021-02277-0.

## Background

Shigellosis or blood dysentery is widespread in underdeveloped or developing regions with poor hygiene and limited access to clean drinking water and has become a serious threat to public health [1, 2]. Shigellosis is caused by nonmotile, facultative anaerobic gram-negative bacilli of the Enterobacteriaceae family, including *S. dysenteriae*, *S. flexneri*, *S. boydii*, and *S. sonnei* [3–5]. *Shigella* species have high effectiveness in invasive systems that enable bacteria to invade and multiply within the human intestinal epithelia, ultimately leading to severe inflammatory colitis, which is referred to as bacillary dysentery or shigellosis [4].

Various virulence factors located on chromosomes or large virulence *inv* plasmids are recognized as crucial factors related to the pathogenesis of shigellosis [6]. Moreover, these different virulence factors are associated with the colonization of intestinal cells and intracellular invasion, which may partly explain why various manifestations are detected in the clinic, such as intestinal inflammatory responses and watery diarrhea [1]. Bacterial cell-to-cell movement and dissemination within epithelial cells of the intestine are allowed by the *iphH* gene, which is encoded by chromosomal DNA and/or recombinant plasmids, while *ial*, which is encoded by plasmids (invasion-associated loci), enables *Shigella* bacteria to penetrate intestinal epithelial tissues [7, 8]. The chromosomal genes *set1A* and *set1B* encode *Shigella* enterotoxin 1 (*ShET-1*) [9, 10], which is easily detected in all *S. flexneri* 2a isolates. *Shigella* enterotoxin 2, which is encoded by the gene *sen*, is located on a large plasmid associated with the virulence of *Shigella* and is found in most *Shigella* of different serotypes and in enteroinvasive *Escherichia coli* (EIEC) [11, 12]. In addition to their enterotoxic activity, *ShET-1* and *ShET-2* play significant roles in the transport of electrolytes and water in the intestine [12]. *VirA* located on large virulence plasmids has a great impact on intercellular spreading and invasion [13]. On the other hand, the type III secretion system (T3SS) is regarded as an important component for bacterial entry and is also composed of several proteins, including a needle-shaped oligomer anchored in the protein complex that connects the inner and outer bacterial membranes. The tip of the needle is an oligomer composed of the invasion plasmid antigens *ipaB*, *ipaC*, and *ipaD* [14–16]. Furthermore, the upstream *ipaB* region is often used as a marker to detect the *ipaBCD* gene.

The natural hosts of *Shigella* are typically humans and other primates [4], but monkeys, rabbits, calves, fish, chickens and piglets were recently reported to be infected with *Shigella* and are thus considered new hosts [4, 17–21]. In recent years, *S. dysenteriae*, *S. flexneri*, and *S. sonnei* have been isolated from cows. Although *Shigella* is becoming a major threat to animals, there is limited information on the genetic background of the isolated strains. Therefore, to identify molecular genotypes and determine the genetic relatedness diversity of local *S. flexneri* strains, we performed analyses using the multilocus sequence typing (MLST), multilocus variable number tandem repeat analysis (MLVA) and pulsed field gel electrophoresis (PFGE) methods.

## Results

### Biochemical characterization

A total of 54 *S. flexneri* from six serotypes, 1a (*n* = 5), 2a (*n* = 26), 2b (*n* = 4), 4a (*n* = 6), 6 (*n* = 8), and Xv (*n* = 5), were analyzed in this study. Based on the results of the biochemical reaction assays, we observed that all 54 *S. flexneri* isolates possessed 4 typical *Shigella* biochemical characteristics (Table 1). Among these BTs (biochemical types), BT4 (the ability to ferment glucose, mannitol, arabinose, and melibiose) was the predominant biotype, accounting for 70.37 % (38/54) of all BTs. Furthermore, BT4 was widely found in each serotype, except serotype 6. *S. flexneri* 2a was distributed among all four biochemical phenotypes and was mainly found in BT4 (22/26, 84.62 %). However, the other five serotype strains had only one or two biochemical phenotypes.

**Table 1:** Biochemical characteristics of *S.flexneri* isolates

Biotype		Total (*n* = 54)	Isolates					
			1a (*n* = 5)	2a (*n* = 26)	2b (*n* = 4)	4a(*n* = 6)	6 (*n* = 8)	Xv (*n* = 5)
BT1	glucose+, mannose+, arabinose-, melibiose+	2 (3.70 %)	0	2(7.69 %)	0	0	0	0
BT2	glucose+, mannose+, arabinose+, melibiose-	9 (16.67 %)	0	1(3.85 %)	0	0	8(100 %)	0
BT3	glucose+, mannose-, arabinose+, melibiose+	5(9.26 %)	0	1(3.85 %)	0	4(66.67 %)	0	0
BT4	glucose+, mannose+, arabinose+, melibiose+	38(70.37 %)	5 (100 %)	22(84.62 %)	4(100 %)	2(33.33 %)	0	5(100 %)

*BT* Biochemical Types

### Virulence factors

**Fig. 1:**
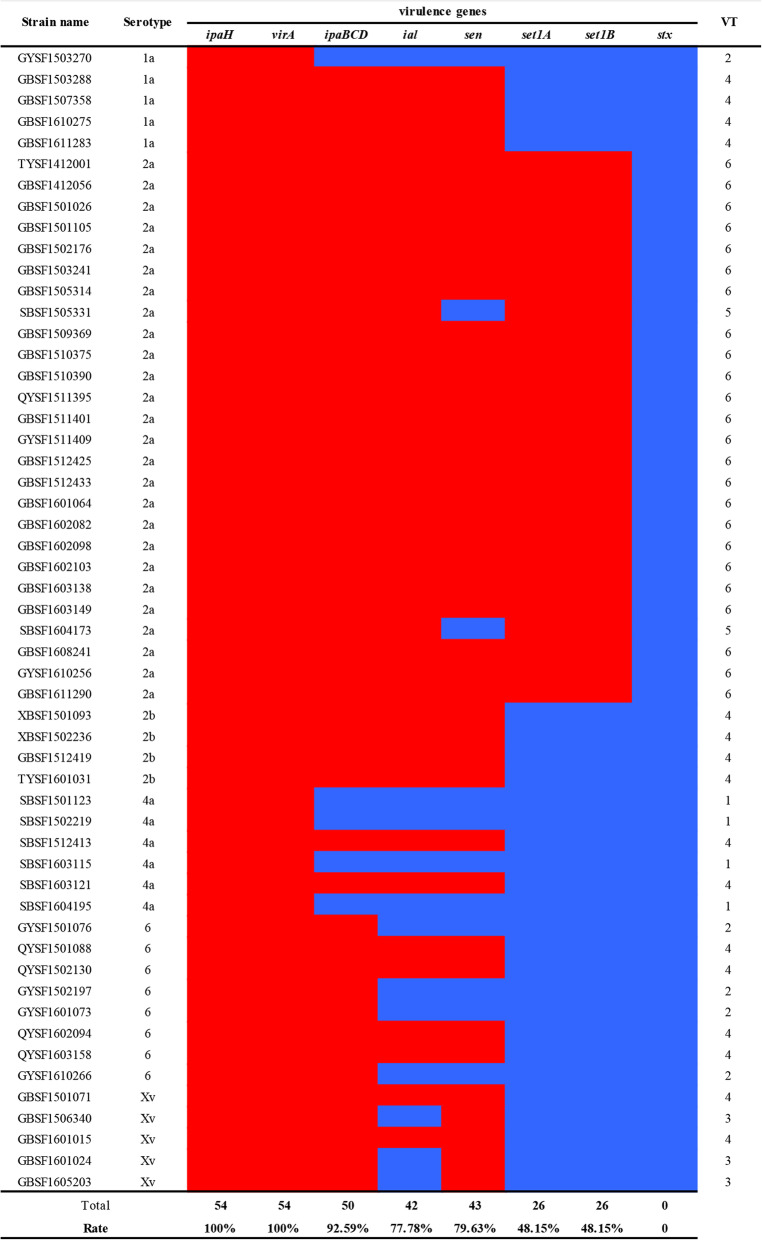
Presence of virulence factors in *S. flexneri* isolates. Red = present; blue = absent. VT: virulence gene profile type

The frequencies of the virulence factor profiles of the *S. flexneri* isolates are listed in Fig. 1. A total of seven virulence factors, including *ipaH* (100 %), *virA* (100 %), *ipaBCD* (92.59 %), *ial* (77.78 %), *sen* (79.63), *set1A* (48.15 %) and *set1B* (48.15 %), were detected in those isolates. None of the studied strains possessed the *stx* gene. The *Shigella* enterotoxin genes *set1A* and *set1B* were present only in *S. flexneri* 2a, and all of these serotype isolates were positive for these two genes.

Regarding the differences in the distributions of the virulence factors, the 54 *S. flexneri* isolates fell into seven virulence gene profile types (VTs) (Table 2). Among these VTs, VT4 (positive for *ipaH*, *virA*, *ipaBCD*, *ial*, and *sen*) and VT6 (positive for *ipaH*, *virA*, *ipaBCD*, *ial*, *sen*, *set1A*, and *set1B*) were the most common, accounting for 29.63 and 44.44 % of all VTs, respectively. Furthermore, 92.59 % of the isolates carried two or more virulence factors. In addition, the virulence factor types were associated with the *S. flexneri* serotype. VT1 was found only in 4a, and VT4 was present in isolates from each serotype, except 2a. *S. flexneri* 2a major belonged to VT6 (24/26, 92.31 %).

**Table 2:** Statistical the rate of each virulence genes types in *S.flexneri isolates*

Virulence genes types		Total (*n* = 54)	Serotype distribution					
			1a (*n* = 5)	2a (*n* = 26)	2b (*n* = 4)	4a (*n* = 6)	6 (*n* = 8)	Xv (*n* = 5)
VT1	*ipaH + virA* + *ipaBCD-ial-sen-set1A-set1B*-	4 (7.41 %)	0	0	0	4 (66.67 %)	0	0
VT2	*ipaH + virA* + *ipaBCD + ial-sen-set1A-set1B*-	5 (9.26 %)	1 (20 %)	0	0	0	4 (50 %)	0
VT3	*ipaH + virA* + *ipaBCD + ial-sen + set1A-set1B*-	3 (5.56 %)	0	0	0	0	0	3 (60 %)
VT4	*ipaH + virA* + *ipaBCD + ial + sen + set1A-set1B*-	16 (29.63 %)	4 (80 %)	0	4 (100 %)	2 (33.33 %)	4 (50 %)	2 (40 %)
VT5	*ipaH + virA* + *ipaBCD + ial + sen-set1A + set1B+*	2 (3.7 %)	0	2 (7.69 %)	0	0	0	0
VT6	*ipaH + virA* + *ipaBCD + ial + sen + set1A + set1B*+	24 (44.44 %)	0	24 (92.31 %)	0	0	0	0

### MLST-based genotype analysis

**Fig. 2:**
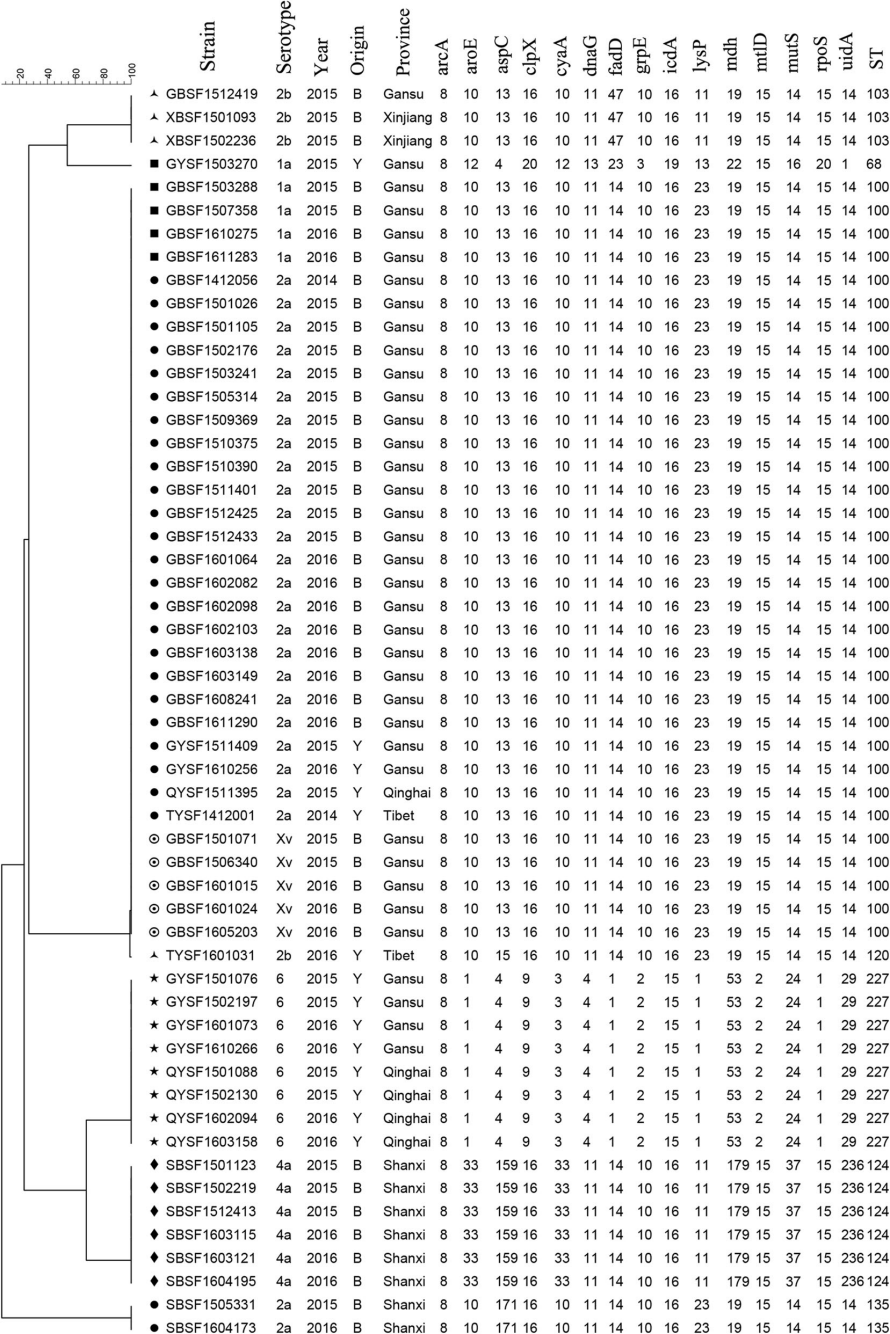
MLST clustering tree of *S. flexneri* isolates isolated from calves with diarrhea during 2014 to 2016. The 54 isolates were analyzed by 15-allele MLST as described in the Materials and Methods. The scale bar in the top corner of the figure represents the similarity of each strain

**Fig. 3:**
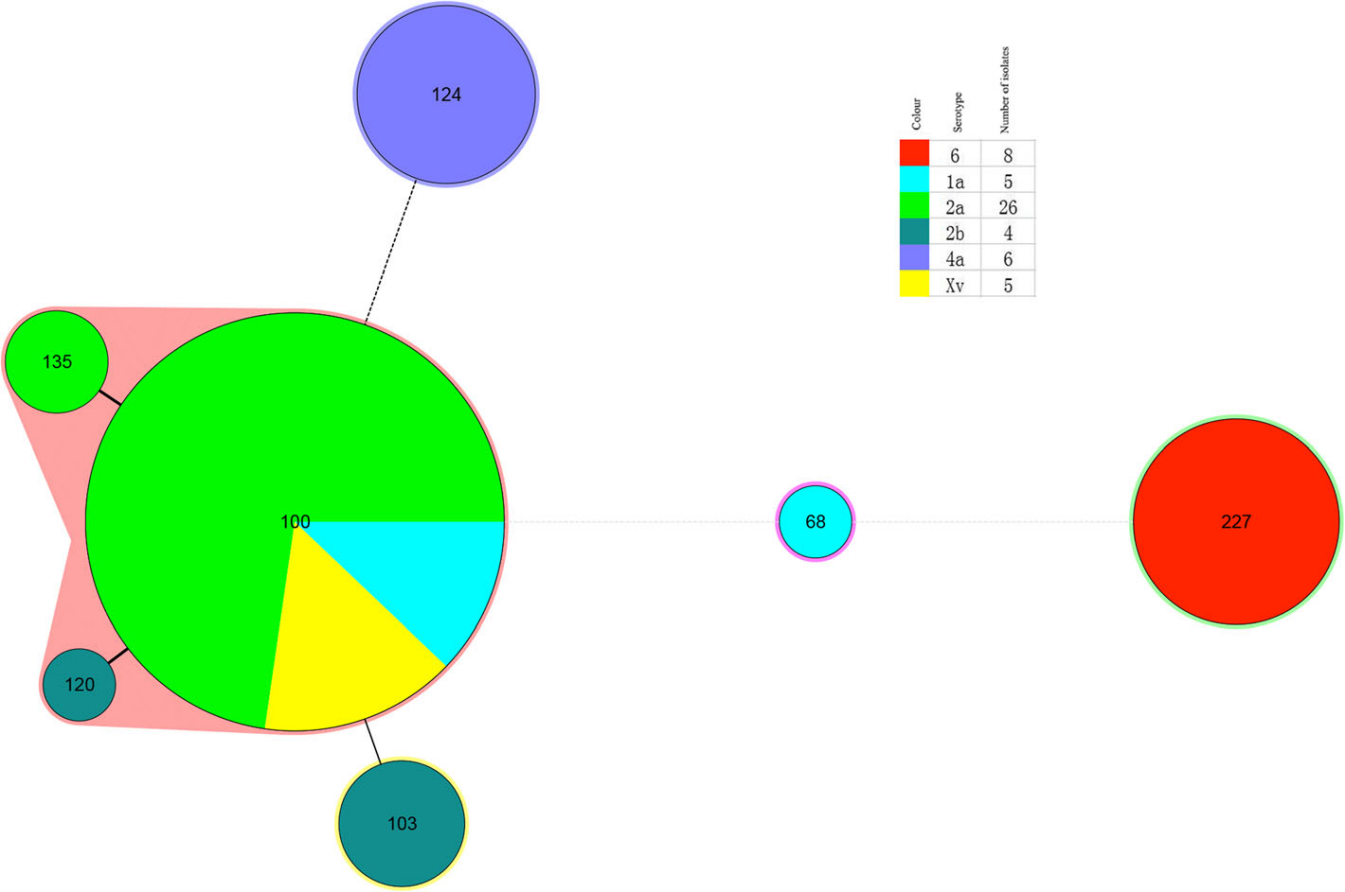
Minimum spanning tree of the 54 *S. flexneri* isolates from calves with diarrhea based on multilocus sequence typing (MLST). The minimum spanning tree was constructed using the 7 identified STs obtained from the 54 isolates using BioNumerics software. Each circle corresponds to a single ST. The shaded zones in different colors correspond to different serotypes. The size of the circle is proportional to the number of isolates, and the color within the circles represents the serotype of the isolates. The corresponding color, serotype, number of isolates and background information are shown to the right of the minimum spanning tree

MLST was performed to analyze the genotypic diversity of *S. flexneri* isolates based on 15 housekeeping genes. The 54 isolates were divided into seven STs: ST68, ST100, ST103, ST120, ST124, ST135 and ST227. Among them, ST227 was novel, while six other STs have previously been reported. These seven STs belonged to several clonal complexes (CCs): CC10 (ST100 and ST103), CC26 (ST68), and others (ST120, ST124, ST135 and ST227). The clustering tree (Fig. 2) based on the MLST data showed that ST68 was a singleton type and that the other six STs contained two or more isolates. The most common ST was ST100 (*n* = 33, 61.11 %), which included isolates of serotypes 1a, 2a, and Xv. All the isolates of ST124 and ST227 belonged to *S. flexneri* 6 and 4a, respectively. The cluster tree indicated that isolates belonging to the same serotype were closely clustered based on the province of isolation. In addition, according to the minimum spanning tree (MST) based on the allele, ST100, ST120 and ST135 had closer relationships and differed only in *aspC* (aspartate aminotransferase), whereas ST68, ST124 and ST227 were very different from the other STs (Fig. 3).

## MLVA-based genotype analysis

**Fig. 4:**
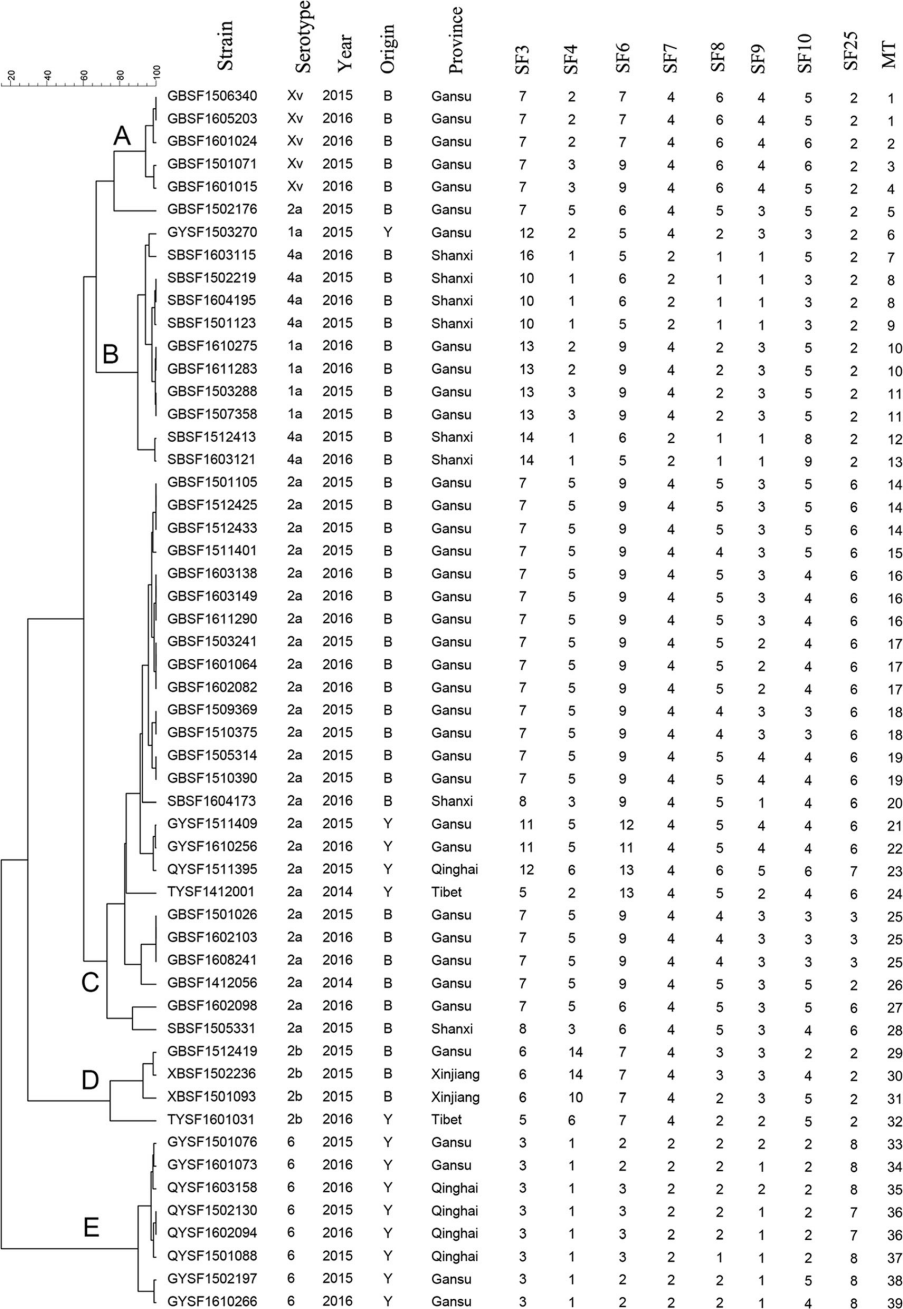
Relationship of *S. flexneri* isolates isolated from calves with diarrhea based on MLVA. Isolates were analyzed using an eight-VNTR locus MLVA scheme. A dendrogram was constructed using UPGMA. The corresponding MLVA types with the copy numbers of the eight VNTRs, serotype, and background information are shown to the right of the dendrogram. The letters A-E represent 5 clusters

MLVA based on eight VNTR loci was performed to further characterize the isolated *S. flexneri* strains. The copy numbers of the eight VNTR loci are listed in Fig. 4. Overall, the 54 isolates based on their unique MLVA profiles were discriminated into 39 different MLVA types (MTs). Among them, twenty-eight MTs belonged to the singleton type, and the other ten MTs contained no more than three isolates. The MLVA cluster tree of the isolates showed that they were divided into five clusters, designated A to E, with a low coefficient of similarity from 20 to 60 % (Fig. 4). Each cluster was further divided into many subclusters. MLVA can cluster different serotype strains separately and distinguish between the same serotype strains. The main cluster, cluster C, was observed to cluster *S. flexneri* 2a isolates and further divided into 15 MTs. Additionally, clusters A (except GBSF1502176), D and E clustered only the Xv, 2b, and 6 serotype strains, respectively. The results showed differences based on geographical origin and time span in the same serotype.

### PFGE-based genotype analysis

**Fig. 5:**
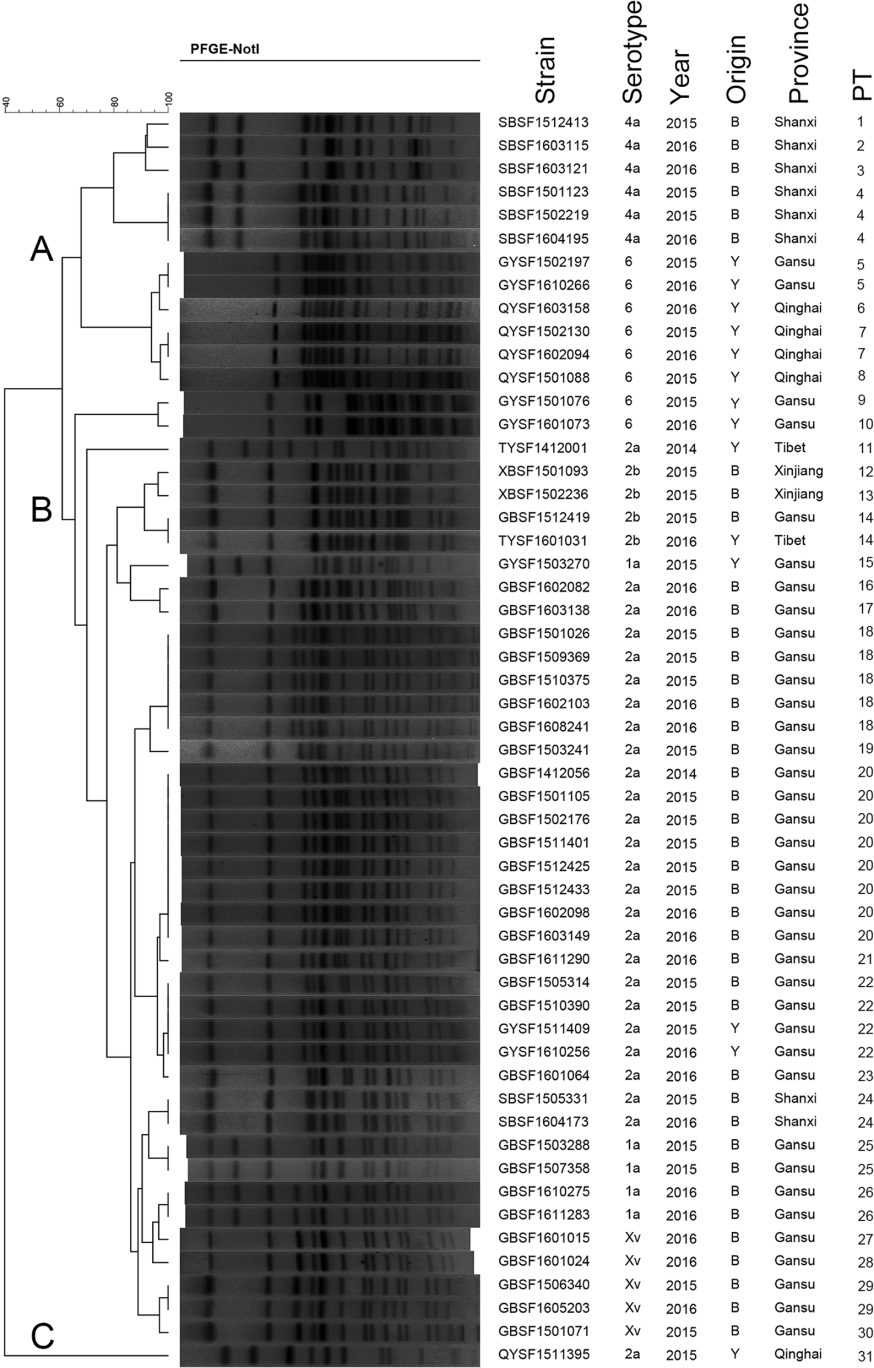
Dendrogram of 54 *Not*I-digested *S. flexneri* isolates based on cluster analysis of PFGE patterns. A dendrogram was constructed using the UPGMA clustering method. The corresponding antibiotic resistance profile, PFGE pattern and background information for each strain are listed on the right side of the dendrogram. PT: PFGE type

The genotypes and genetic relatedness diversity of 54 *S. flexneri* isolates were assessed by PFGE. *Not*I-digested *S. flexneri* chromosomal DNA generated 31 reproducible unique PFGE patterns (PTs), each with 11–16 bands (Fig. 5). Eleven patterns were represented by more than one isolate, with PT20 (n = 8) containing the most isolates, followed by PT18 (*n* = 5). The dendrogram of *S. flexneri* isolates showed low similarity (40-60 %) and could be classified into three gross clusters on the basis of their serotypes: clusters A, B and C. Isolates belonging to the same serotype but recovered in different years showed clear relatedness, as indicated by their grouping in the same clusters. The majority of serotype 2a isolates, with the exception of isolate QYSF1511395, grouped together in cluster B. The QYSF1511395 strain isolated from Qinghai Province clustered independently in cluster C. Isolates 1a, 2b and Xv clustered into cluster B and were closely related to the serotype 2a isolates. However, the isolates of serotypes 4a and 6 were assigned to cluster A with a relatively close relationship, but different serotype strains clustered separately.

## Discussion

*Shigella* is an important invasive enteric infectious pathogen known for its sporadic, epidemic and pandemic spread [3],remains still a landmark cause of inflammatory diarrhea and dysentery thus poses a serious challenge to public health and is particularly tracked in most middle-income countries and regions with substandard hygiene and poor-quality water supplies [22]. All four types of *Shigella* can cause shigellosis, but *S. flexneri* is the most common bacterial preparation used for shigellosis [23]. The traditional hosts of this pathogen are limited to primates; however, the range of hosts has been extended to many animals in recent decades [4]. The symptoms of shigellosis in humans include diarrhea (100 %), headache (100 %), fever (100 %), nausea (99 %), abdominal cramping (97 %), vomiting (95 %), and bloody stools (51 %); however, the symptoms in animals are unclear [24]. A better understanding of the hosts of *Shigella* is needed to assess their potential effects on animal health; otherwise, preventing *Shigella* from causing disease is a challenge.

The pathogenesis of *Shigella* contributes to the organism’s ability to invade, replicate and spread intercellularly within the colonic epithelium. Pathogenic factors cause pathogenic Shigella to invade intestinal epithelial cells, leading to dysentery and other intestinal clinical symptoms in the host [25], and its pathogenesis is often multifactorial and coordinated [26]. Virulence factors have become important indicators of pathogenic bacteria.

Based on the detection of virulence factors, the *Shigella* isolates used in the present study had vast genetic diversity. Our results showed that *ipaH* and *virA* were found in each strain. Arabshahi et al. similarly showed that *ipaH* was present in all *Shigella* isolates and that *virA* was harbored by 88.9 % (8/9) of *S. flexneri isolates* [27], which agrees with a previous study that demonstrated that *ipaH* is carried by all four *Shigella* species as well as by enteroinvasive *E. coli* (EIEC). Multiple copies (*ipaH*1.4, *ipaH*2.5, *ipaH*4.5, *ipaH*7.8 and *ipaH*9.8) on large plasmids and chromosomes may explain why the *ipaH* gene tested positive in all isolates. Therefore, as a diagnostic tool for detecting *Shigella*, the *ipaH* gene is often an appealing target, even in the absence of a plasmid [28]. *VirA* was initially thought to invade *Shigella*; however, a structural analysis showed that *VirA* lacks papain-like protease activity to promote tubulin division. *VirA* belongs to the GTPase-activating protein family, which is involved in the cleavage of a single membrane into vacuoles. Previous studies have shown that *VirA* is often present in *Shigella* and is an important terminal point for bacteria to invade host cells and nucleate actin at one end of bacteria [9, 29].

Expert opinions have suggested that the T3SS is essential for host cell invasion and intracellular survival among virulence factors, whereas *IpaB*, *IpaC*, and *IpaD* are key factors of virulent *Shigella* [9, 30, 31]. Unlike the *ipaH* gene, the *ial* gene is not common. The *ipaH* gene is located only on the *inv* plasmid, and compared with the chromosome gene, the stability of the IPAH plasmid for storage/subculturing is poor [6–8]. Our results show that the *ial* gene has high invasiveness in the isolates studied. Therefore, it should be noted that the *ial* gene is involved in the invasion of intestinal cells and that the higher positivity rate of this gene in *S*. *flexneri* might indicate stronger aggressiveness.

The *Shigella* enterotoxins *ShET-1* and *ShET-2*, which alter electrolyte and water transport in the small intestine, can cause diarrhea and dehydration [22]. *ShET-1* is located on chromosomes encoding *set1* (A and B subunit) genes, is almost exclusively found in several *S*. *flexneri* serotype 2 isolates and is rarely found in other serotypes [32]. Consistent with previous studies, our study showed that *set1A* and *set1B* were detected only in the *S. flexneri* 2a strain. The plasmid encoding *ShET-2* (encoded by *sen*) is an enterotoxin hemolysin that causes an inflammatory response during Shigella invasion [12, 22]. It has been reported that there is a close relationship among *sen*, *set* enterotoxins and bloody diarrhea [22], which implies that *sen* and *set* enterotoxins are pathogenic factors of bloody diarrhea. However, unlike *ShET-1*, *ShET-2* could be harbored by other species of *Shigella.*

The molecular characterization of strains is significant for epidemiological studies. However, few reports are available to systematically understand the molecular characteristics of *S. flexneri* isolated from animals. Several useful genotyping tools with higher discriminatory power than traditional tests, including MLST [33], PFGE [34] and MLVA [35], have recently been applied to explore and analyze the characteristics of *Shigella* isolates. Phylogenetic analysis, as an important method for supporting strain isolation, is based on differences in strain genetics.

MLST is an important source of sequence data for relative genetics and thus provides a tool for exploring molecular evolutionary methods among bacteria [36]. With the key elements of 15 housekeeping genes and analysis of the EcMLST database, the advantage of MLST is the comparison of data from different laboratories. Our results suggested that the predominant ST was ST 100, which has previously been found in human *S. flexneri* isolates [37, 38]. Specifically, isolates belonging to the same serotype often showed one ST type, indicating the low discriminative ability of closely related strains within a specific serotype due to the high sequence conservation of housekeeping genes.

Compared with MLST profiles, MLVA and PFGE may be forceful tools that can provide a satisfactory level of discrimination. However, the function of MLVA in the phylogenetic analysis of different bacterial species or serotypes is poorly targeted [39]. Nevertheless, MLVA is an ordinarily used typing tool that has been used to establish genetic relatedness and perform phylogenetic analysis among strains of monomorphic species. In our study, with approximately 20 % similarity, the 54 *S. flexneri* isolates were divided into 39 different MTs and clustered into 5 groups. Previous studies have also shown the high resolving power of MLVA in closely related strains [40–42]. Though applied in a limited collection of *S. flexneri* isolates, this study indicates the high discriminatory power of the MLVA method for subtyping strains with the same serotype.

With its strong function and widespread use, PFGE is also an applicable typing tool available in the laboratory for discriminating several enteric bacteria, such as *Shigella*. PFGE has a high degree of intra- and interlaboratory reproducibility when standardized protocols are followed [43]. Thirty-one low homophyly and unique PFGE patterns confirmed the existence of diverse *S. flexneri* clones and the usefulness of PFGE in local epidemiological studies.

## Conclusions

This study demonstrated that spontaneously prevalent *S. flexneri* in cows shelter the same virulence factors as the prevalent isolates in humans. Therefore, these isolates are a potential threat to public safety. To systematically understand *S. flexneri*, the PFGE, MLVA and MLST methods were applied to characterize the 54 isolates hereditary. MLVA based on 8 VNTR loci discriminated the 54 isolates into 39 different MTs, PFGE based on *Not*I digestion ambiguously differentiated the 54 isolates into 31 PTs, MLST based on 15 housekeeping genes differentiated the 54 isolates into 7 STs, and 1 ST (ST227) was novel. Although MLST provided suitable discrimination in *S. flexneri* subtyping, PFGE and MLVA might both exhibit a higher discriminatory ability. Overall, the data from this study will provide a useful typing resource, which will provide a scientific basis for addressing clinical and epidemiological issues regarding *S. flexneri*.

## Methods

### Bacterial isolates and bacteriological examination

Animal-based active surveillance was conducted in 3321 calves with diarrhea from five provinces (Gansun, Shanxi, Qinghai, Xinjiang and Tibet) in northwestern China from 2014 to 2016. All of the isolates were collected directly from fresh stool samples following plating on *Salmonella-Shigella* (SS) selective agar and confirmation on MacConkey (MAC) agar at 37°C for 24 h. Colorless, semitransparent, smooth, and moist circular plaques were considered presumptive *Shigella* for biochemical confirmation. Biochemical tests were performed on *S. flexneri* using API20E test strips (bioMerieux Vitek, Marcy-l’ Etoile, France), and the serotype was tested by a commercially available agglutinating antibody kit (Denka Seiken, Tokyo, Japan) according to the manufacturers’ recommendations. Information on the *S. flexneri* isolates in this study is listed in Figs. 2 , 4 and 5.

### Preparation of DNA templates

The DNA templates for PCR (virulence factors, MLST, MLVA) were directly extracted from bacterial colonies using the boiled lysate method as previously reported [44].

### Detection of virulence factors

All 54 strains were tested by PCR for the presence of 8 virulence-associated genes, namely, *ipaH*, *ipaBCD*, *virA*, *ial*, *stx*, *set1A*, *set1B*, and *sen*, according to published procedures [15, 45, 46]. PCRs were performed according to published protocols, and the primer sequences are listed in Table S1.

### Multilocus sequence typing

All isolates were subjected to MLST according to the protocols described in the EcMLST database (http://www.shigatox.net/ecmlst). The PCR products were bidirectionally sequenced, and the sequences of the 15 housekeeping genes were edited by using SeqMan 7.0. Each unique allele was assigned a different number, and the allelic profile (string of fifteen allelic loci) was used to define the ST of each isolate [47]. Clustering and minimum spanning tree (MST) analyses were used to infer relationships among the isolates using the fingerprint analysis software BioNumerics (version 7.1).

### Multilocus variable number tandem repeat analysis

MLVA of 8 VNTR loci (SF3, SF4, SF6, SF7, SF8, SF9, SF10 and SF25) was performed using a previously described method [35]. The forward primer for each primer set was labeled at its 5’ end with the ABI-compatible dyes HEX, 6’-FAM, TAMRA, and ROX (Table S2). In these cases, the loci were individually amplified, with each 20 µL PCR mixture containing 1 µL of each primer, 1 µL of DNA template, 10 µL of Taq MasterMix (Takara, Japan) and deionized water to a final volume of 20 µL. PCR was performed with a denaturing step at 94 °C for 5 min, followed by 30 cycles of amplification at 94 °C for 30 s, 55 °C for 45 s, and 72 °C for 45 s and a final extension at 72 °C for 5 min at the final step.

The PCR products were analyzed by capillary electrophoresis on an ABI Prism 3730 XL Genetic Analyzer with the GeneScan 500 LIZ Size Standard as previously described [48]. The number of repeat units for each allele was calculated from the length of the amplicon. The copy number of each VNTR locus was subjected to cluster analysis using the MST algorithm and the categorical coefficient provided in BioNumerics software. Each unique allelic string was designated a unique MLVA type. A dendrogram was constructed by UPGMA clustering based on categorical coefficient analysis [35, 49].

### Pulsed-field gel electrophoresis

DNA fingerprinting was performed by PFGE with the restriction enzyme *Not*I (TaKaRa; Japan) according to the international standards set by the CDC. PFGE images were photographed with a Universal Hood II (Bio-Rad; USA) and analyzed with BioNumerics using the Dice similarity coefficient, unweighted pair-group method with the arithmetic mean (UPGMA) and 1.0 % band position tolerance. A PFGE type (PT) was defined as a pattern with one or more DNA bands different from other patterns.

## Supplementary Information

**Additional file 1: Table S1.** Primers used for the detection of virulence genes.

**Additional file 2: Table S2.** MLVA primers for *S. flexneri*.

## Data Availability

The data supporting the findings of this study are contained within the manuscript.

## Abbreviations

VTs: Virulence gene profile types
BTs: Biochemical types
MLVA: Multiple-locus variable number tandem repeat analysis
VNTR: Variable number of tandem repeats
PFGE: Pulsed-field gel electrophoresis
MLST: Multilocus sequence typing

## References

[1] Dutta S, Jain P, Nandy S, Matsushita S, Yoshida S (2014). Molecular characterization of serologically atypical provisional serovars of Shigella isolates from Kolkata, India. J Med Microbiol.

[2] Soltan Dallal MM, Ranjbar R, Pourshafie MR (2011). The study of antimicrobial resistance among Shigella flexneri strains isolated in Tehran, Iran. J Pediatr Infect Dis.

[3] Ranjbar R, Bolandian M, Behzadi P (2017). Virulotyping of Shigella spp. isolated from pediatric patients in Tehran, Iran. Acta Microbiol Immunol Hung.

[4] Shi R, Yang X, Chen L, Chang HT, Liu HY, Zhao J (2014). Pathogenicity of Shigella in chickens. PLoS One..

[5] Ranjbar R, Behnood V, Memariani H, Najafi A, Moghbeli M, Mammina C (2016). Molecular characterisation of quinolone-resistant Shigella strains isolated in Tehran, Iran. J Glob Antimicrob Resist.

[6] Shen Y, Qian H, Gong J, Deng F, Dong C, Zhou L (2013). High prevalence of antibiotic resistance and molecular characterization of integrons among Shigella isolates in Eastern China. Antimicrob Agents Chemother.

[7] Phantouamath B, Sithivong N, Insisiengmay S, Ichinose Y, Higa N, Song T (2005). Pathogenicity of Shigella in healthy carriers: a study in Vientiane, Lao People’s Democratic Republic. Jpn J Infect Dis.

[8] Ashida H, Sasakawa C (2016). Shigella IpaH Family Effectors as a Versatile Model for Studying Pathogenic Bacteria. Front Cell Infect Microbiol..

[9] Schroeder GN, Hilbi H (2008). Molecular pathogenesis of Shigella spp.: controlling host cell signaling, invasion, and death by type III secretion. Clin Microbiol Rev.

[10] Sousa MÂ, Mendes EN, Collares GB, Péret-Filho LA, Penna FJ, Magalhães PP (2013). Shigella in Brazilian children with acute diarrhoea: prevalence, antimicrobial resistance and virulence factors. Mem Inst Oswaldo Cruz.

[11] Farfán MJ, Toro CS, Barry EM, Nataro JP (2011). Shigella enterotoxin-2 is a type III effector that participates in Shigella-induced interleukin 8 secretion by epithelial cells. FEMS Immunol Med Microbiol.

[12] da Cruz CB, de Souza MC, Serra PT, Santos I, Balieiro A, Pieri FA (2014). Virulence factors associated with pediatric shigellosis in Brazilian Amazon. Biomed Res Int.

[13] Zaidi MB, Estrada-García T (2014). Shigella: a highly virulent and elusive pathogen. Curr Trop Med Rep.

[14] Büttner D, Bonas U (2006). Who comes first? How plant pathogenic bacteria orchestrate type III secretion. Curr Opin Microbiol.

[15] Faruque SM, Khan R, Kamruzzaman M, Yamasaki S, Ahmad QS, Azim T (2002). Isolation of Shigella dysenteriae type 1 and S. flexneri strains from surface waters in Bangladesh: comparative molecular analysis of environmental Shigella isolates versus clinical strains. Appl Environ Microbiol.

[16] Marteyn B, Gazi A, Sansonetti P (2012). Shigella: a model of virulence regulation in vivo. Gut Microbes.

[17] Onyango DM, Wandili S, Kakai R, Waindi EN (2009). Isolation of Salmonella and Shigella from fish harvested from the Winam Gulf of Lake Victoria, Kenya. J Infect Dev Ctries.

[18] Priamukhina NS, Kilesso VA, Tikhomirov ED. Animal carriers of Shigella and their possible epidemiological importance. Zh Mikrobiol Epidemiol Immunobiol. 1984;11:20–4. 6441399

[19] Maurelli AT, Routh PR, Dillman RC, Ficken MD, Weinstock DM, Almond GW (1998). Shigella infection as observed in the experimentally inoculated domestic pig, Sus scrofa domestica. Microb Pathog.

[20] Zhu Z, Cao M, Zhou X, Li B, Zhang J (2017). Epidemic characterization and molecular genotyping of Shigella flexneri isolated from calves with diarrhea in Northwest China. Antimicrob Resist Infect Control.

[21] Zhu Z, Shi Y, Zhou X, Li B, Zhang J (2018). Molecular characterization of fluoroquinolone and/or cephalosporin resistance in Shigella sonnei isolates from yaks. BMC Vet Res.

[22] Gu B, Fan W, Qin T, Kong X, Dong C, Tan Z (2019). Existence of virulence factors in clinical Shigella sonnei isolates from Jiangsu Province of China: a multicenter study. Ann Transl Med.

[23] Sangeetha AV, Parija SC, Mandal I, Krishnamurthy S (2014). Clinical and microbiological profiles of shigellosis in children. J Health Popul Nutr.

[24] Ranjbar R, Hosseini MJ, Kaffashian AR (2010). An outbreak of shigellosis due to Shigella flexneri serotype 3a in a prison in Iran. Arch Iran Med.

[25] Ashida H, Ogawa M, Mimuro H, Kobayashi T, Sanada T, Sasakawa C (2011). Shigella are versatile mucosal pathogens that circumvent the host innate immune system. Curr Opin Immunol.

[26] Qu M, Zhang X, Liu G, Huang Y, Jia L, Liang W (2014). An eight-year study of Shigella species in Beijing, China: serodiversity, virulence factors, and antimicrobial resistance. J Infect Dev Ctries.

[27] Arabshahi S, Novinrooz A, Ranjbar R (2020). Molecular characterization of Shigella species isolated from diarrheal patients in Tehran, Iran: phylogenetic typing and its association with virulence gene profiles and a novel description of Shigella invasion associated locus. Eur J Clin Microbiol Infect Dis.

[28] Vu DT, Sethabutr O, Von Seidlein L, Tran VT, Do GC, Bui TC (2004). Detection of Shigella by a PCR assay targeting the ipaH gene suggests increased prevalence of shigellosis in Nha Trang, Vietnam. J Clin Microbiol.

[29] Yaghoubi S, Ranjbar R, Dallal MMS, Fard SY, Shirazi MH, Mahmoudi M (2017). Profiling of virulence-associated factors in Shigella species isolated from acute pediatric diarrheal samples in Tehran, Iran. Osong Public Health Res Perspect.

[30] Parsot C (2009). Shigella type III secretion effectors: how, where, when, for what purposes?. Curr Opin Microbiol.

[31] Mattock E, Blocker AJ (2017). How do the virulence factors of Shigella work together to cause disease?. Front Cell Infect Microbiol..

[32] Roy S, Thanasekaran K, Dutta Roy AR, Sehgal SC (2006). Distribution of Shigella enterotoxin genes and secreted autotransporter toxin gene among diverse species and serotypes of shigella isolated from Andaman Islands, India. Trop Med Int Health.

[33] Cao Y, Wei D, Kamara IL, Chen W (2012). Multi-Locus Sequence Typing (MLST) and Repetitive Extragenic Palindromic Polymerase Chain Reaction (REP-PCR), characterization of shigella spp. over two decades in Tianjin China. Int J Mol Epidemiol Genet.

[34] Xia S, Xu B, Huang L, Zhao JY, Ran L, Zhang J (2011). Prevalence and characterization of human Shigella infections in Henan Province, China, in 2006. J Clin Microbiol..

[35] Wang YW, Watanabe H, Phung DC, Tung SK, Lee YS, Terajima J (2009). Multilocus variable-number tandem repeat analysis for molecular typing and phylogenetic analysis of Shigella flexneri. BMC Microbiol.

[36] Hu Y, Xi Z, Liu X, Wang J, Guo Y, Ren D (2020). Identification and molecular characterization of Wolbachia strains in natural populations of Aedes albopictus in China. Parasit Vectors.

[37] Li S, Sun Q, Wei X, Klena JD, Wang J, Liu Y (2015). Genetic characterization of Shigella flexneri isolates in Guizhou Province, China. PLoS One.

[38] Cui X, Wang J, Yang C, Liang B, Ma Q, Yi S (2015). Prevalence and antimicrobial resistance of Shigella flexneri serotype 2 variant in China. Front Microbiol..

[39] Cheng T, Shi X, Yong W, Wang J, Xie G, Ding J (2014). Molecular typing of Shigella sonnei isolates circulating in Nanjing, China, 2007–2011. J Infect Dev Ctries.

[40] Filliol-Toutain I, Chiou CS, Mammina C (2011). Global distribution of Shigella sonnei clones. Emerg Infect Dis.

[41] Ranjbar R, Memariani M, Memariani H (2015). Diversity of variable number tandem repeat Loci in Shigella species isolated from pediatric patients. Int J Mol Cell Med.

[42] Ranjbar R, Memariani M (2015). Multilocus variable-number tandem-repeat analysis for genotyping of Shigella sonnei strains isolated from pediatric patients. Gastroenterol Hepatol Bed Bench.

[43] Ribot EM, Fair MA, Gautom R, Cameron DN, Hunter SB, Swaminathan B (2006). Standardization of pulsed-field gel electrophoresis protocols for the subtyping of Escherichia coli O157:H7, Salmonella, and Shigella for PulseNet. Foodborne Pathog Dis.

[44] Ahmed AM, Shimamoto T, Shimamoto T (2013). Molecular characterization of multidrug-resistant avian pathogenic Escherichia coli isolated from septicemic broilers. Int J Med Microbiol.

[45] Das A, Natarajan M, Mandal J (2016). The emergence of quinolone resistant Shigella sonnei, Pondicherry, India. PLoS One.

[46] Vargas M, Gascon J, Jimenez De Anta MT, Vila J (1999). Prevalence of Shigella enterotoxins 1 and 2 among Shigella strains isolated from patients with traveler’s diarrhea. J Clin Microbiol.

[47] Schaumburg F, Alabi AS, Kaba H (2015). Molecular characterization of Shigella spp. from patients in Gabon 2011–2013. Trans R Soc Trop Med Hyg.

[48] Liang SY, Watanabe H, Terajima J (2007). Multilocus variable-number tandem repeat analysis for molecular typing of Shigella sonnei. J Clin Microbiol..

[49] Hyytia-Trees E, Smole SC, Fields PA, et al. Second generation subtyping: a proposed PulseNet protocol for multiple-locus variable-number tandem repeat analysis of Shiga toxin-producing Escherichia coli O157 (STEC O157). Foodborne Pathog Dis. 2006;3:118–131. 10.1089/fpd.2006.3.118 16602987

