# Supplementary material for: Virulence factors and molecular characteristics of Shigella flexneri isolated from calves with diarrhea
Source: BMC Microbiol. 2021 Jul 16;21:214. doi: 10.1186/s12866-021-02277-0 (PMC8285881; doi:10.1186/s12866-021-02277-0)
Supplement: Supplementary file 1 — Additional file 1: Table S1. Primers used for the detection of virulence genes. [file 12866_2021_2277_MOESM1_ESM.docx]

Table S1 Primers for detection of virulence genes.

| Target | Primer sequence | Amplicon size | Reference |
| --- | --- | --- | --- |
| *ipaH* | F: TGGAAAAACTCAGTGCCTCT | 423 | Das A et al., 2016 |
|  | R: CCAGTCCGTAAATTCATTCT |  |  |
| *virA* | F: CTGCATTCTGGCAATCTCTTCACATC | 215 | Vargas M et al., 1999 |
|  | R:TGATGAGCTAACTTCGTAAGCCCTCC |  |  |
| *ipaBCD* | F: GCTATAGCAGTGACATG | 500 | Faruque SM et al., 2002 |
|  | R: ACGAGTTCGAAGCACTC |  |  |
| *ial* | F: CTGGATGGTATGGTGAGG | 320 | Das A et al., 2016 |
|  | R: GGAGGCCAACAATTATTTCC |  |  |
| *sen* | F: ATGTGCCTGCTATTATTTAT | 799 | Das A et al., 2016 |
|  | R: CATAATAATAAGCGGTCAGC |  |  |
| *Set1A* | F: TCACGCTACCATCAAAGA | 309 | Das A et al., 2016 |
|  | R: TATCCCCCTTTGGTGGTA |  |  |
| *Set1B* | F: GTGAACCTGCTGCCGATATC | 147 | Das A et al., 2016 |
|  | R: ATTAGTGGATAAAAATGACG |  |  |
| *stx* | F: CAGTTAATGTGGTTGCGAAG | 895 | Vargas M et al., 1999 |
|  | R: CTGCTAATAGTTCTGCGCATC  CTGCTAATAGTTCTGCGCATC |  |  |
