# Supplementary material for: Virulence factors and molecular characteristics of Shigella flexneri isolated from calves with diarrhea
Source: BMC Microbiol. 2021 Jul 16;21:214. doi: 10.1186/s12866-021-02277-0 (PMC8285881; doi:10.1186/s12866-021-02277-0)
Supplement: Supplementary file 2 — Additional file 2: Table S2. MLVA primers for S. flexneri. [file 12866_2021_2277_MOESM2_ESM.docx]

Table S2 The MLVA primers for *S.flexneri.*

| Locus | Primer 5’-3’ | Dye | Core number (bp) | Core sequence |
| --- | --- | --- | --- | --- |
| SF3 | TTTTAGCATGGTTATTCTCCTTG | HEX | 6 | GGTGCA |
|  | TTTTAGCATGGTTATTCTCCTTG |  |  |  |
| SF4 | CCGAGGAACAGTACGCTTTT | 6-FAM | 6 | CCAGCC |
|  | CCTGCTGGCTTACCACACC |  |  |  |
| SF6 | GAAATACTCAGGTGTCAACATTCG | ROX | 9 | TTAATGATT |
|  | GCTTTGGAGAGTATTATTGCCTGT |  |  |  |
| SF7 | CCGGAACTATTGGTCTGGAA | ROX | 5 | CGCAG |
|  | ATCGACCACATGTTCAATGG |  |  |  |
| SF8 | GCCAGAGCTGTTGGTACTGG | TAMRA | 9 | ATCAGCACC |
|  | CGATTTTTGTCCGCTGAAAG |  |  |  |
| SF9 | CAAATGGTAACGTCGCATCA | 6-FAM | 9 | GCCAGTTCA |
|  | ATGGGATTGCTGCGTAACAC |  |  |  |
| SF10 | CGGGAACCGTTTTGTATCA | ROX | 6 | ACCAAT |
|  | AAGGACGCACGTCAAATACC |  |  |  |
| SF25 | GAGCAGGGATCCGTCATTTA | ROX | 5 | GTTAT |
|  | CGTGATGATTTCCGAGGTGT |  |  |  |
